# A framework of evidence-based decision-making in health system management: a best-fit framework synthesis

**DOI:** 10.1186/s13690-022-00843-0

**Published:** 2022-03-29

**Authors:** Tahereh Shafaghat, Peivand Bastani, Mohammad Hasan Imani Nasab, Mohammad Amin Bahrami, Mahsa Roozrokh Arshadi Montazer, Mohammad Kazem Rahimi Zarchi, Sisira Edirippulige

**Affiliations:** 1 School of Management and Medical Informatics, Health Human Recourses Research Center, Shiraz University of Medical Sciences, Shiraz, Iran; 2 Department of Health Care Management, School of Public Health, Health Policy and Management Research Center, Shahid Saoughi University of Medical Sciences, Yazd, Iran; 3 Faculty of Health and Behavioral Sciences, School of Dentistry, University of Queensland, QLD 4072 Brisbane, Australia; 4 Social Determinants of Health Research Center, Lorestan University of Medical Sciences, Khorramabad, Iran; 5 Student Research Committee, School of Management and Medical Informatics, Shiraz University of Medical Sciences, Shiraz, Iran; 6 Faculty of Medicine, Center for Health Services Research, The University of Queensland, Brisbane, Australia

**Keywords:** Evidence-based decision-making, Management, Health system, Best-fit framework synthesis

## Abstract

**Background:**

Scientific evidence is the basis for improving public health; decision-making without sufficient attention to evidence may lead to unpleasant consequences. Despite efforts to create comprehensive guidelines and models for evidence-based decision-making (EBDM), there isn`t any to make the best decisions concerning scarce resources and unlimited needs**.** The present study aimed to develop a comprehensive applied framework for EBDM.

**Methods:**

This was a Best-Fit Framework (BFF) synthesis conducted in 2020. A comprehensive systematic review was done via six main databases including PUBMED, Scopus, Web of Science, Science Direct, EMBASE, and ProQuest using related keywords. After the evidence quality appraisal, data were extracted and analyzed via thematic analysis. Results of the thematic analysis and the concepts generated by the research team were then synthesized to achieve the best-fit framework applying Carroll et al. (2013) approach.

**Results:**

Four thousand six hundred thirteen studies were retrieved, and due to the full-text screening of the studies, 17 final articles were selected for extracting the components and steps of EBDM in Health System Management (HSM). After collecting, synthesizing, and categorizing key information, the framework of EBDM in HSM was developed in the form of four general scopes. These comprised inquiring, inspecting, implementing, and integrating, which included 10 main steps and 47 sub-steps.

**Conclusions:**

The present framework provided a comprehensive guideline that can be well adapted for implementing EBDM in health systems and related organizations especially in underdeveloped and developing countries where there is usually a lag in updating and applying evidence in their decision-making process. In addition, this framework by providing a complete, well-detailed, and the sequential process can be tested in the organizational decision-making process by developed countries to improve their EBDM cycle.

## Background

Globally, there is a growing interest in using the research evidence in public health policy-making [1, 2]. Public health systems are diverse and complex, and health policymakers face many challenges in developing and implementing policies and programs that are required to be efficient [1, 3]. The use of scientific evidence is considered to be an effective approach in the decision-making process [3–5]. Due to the lack of sufficient resources, evidence-based decision-making **(**EBDM) is regarded as a way to optimize costs and prevent wastes [6]. At the same time, the direct consequence of ignoring evidence is poorer health for the community [7].

Evidence suggests that health systems often fail to exploit research evidence properly, leading to inefficiencies, death or reduced quality of citizens’ lives, and a decline in productivity [8]. Decision-making in the health sector without sufficient attention to evidence may lead to a lack of effectiveness, efficiency, and fairness in health systems [9]. Instead, the advantages of EBDM include adopting cost-effective interventions, making optimal use of limited resources, increasing customer satisfaction, minimizing harm to individuals and society, achieving better health outcomes for individuals and society [10, 11], as well as increasing the effectiveness and efficiency of public health programs [12].

Using the evidence in health systems’ policymaking is a considerable challenging issue that many developed and developing countries are facing nowadays. This is particularly important in the latter, where their health systems are in a rapid transition [13]. For instance, although in 2012, a study in European Union countries showed that health policymakers rarely had necessary structures, processes, and tools to exploit research evidence in the policy cycle [14], the condition can be worse among the developing and the underdeveloped ones. For example, evidence-based policy-making in developing countries like those located in the Middle East can have more significant impacts [15, 16]. In such countries resources are generally scarce, so the policymakers' awareness of research evidence becomes more important [17]. In general, low and middle-income countries have fewer resources to deal with health issues and need quality evidence for efficient use of these resources [7].

Since the use of EBDM is fraught with the dilemma of most pressing needs and having the least capacity for implementation especially in developing countries [16], efforts have been made to create more comprehensive guidelines for EBDM in healthcare settings, in recent years [18]. Stakeholders are significantly interested in supporting evidence-based projects that can quickly prioritize funding allocated to health sectors to ensure the effective use of their financial resources [19–21]. However, it is unlikely that the implementation of EBDM in Health System Management (HSM) will follow the evidence-based medicine model [10, 22]. On the other hand, the capacity of organizations to facilitate evidence utilization is complex and not well understood [22], and the EBDM process is not usually institutionalized within the organizational processes [10]. A study in 2005 found that few organizations support the use of research evidence in health-related decisions, globally [23]. Weis et al. (2012) also reported there is insufficient information on EBDM in local health sectors [12]. In general, it can be emphasized that relatively few organizations hold themselves accountable for using research evidence in developing health policies [24]. To the best of our knowledge, there isn`t any comprehensive global and practical model developed for EBDM in health systems/organizations management. Accordingly, the present study aimed to develop a comprehensive framework for EBDM in health system management. It can shed the light on policymakers to access a detailed practical model and enable them to apply the model in actual conditions.

## Methods

This was a Best Fit Framework (BFF) synthesis conducted in 2020 to develop a comprehensive framework for EBDM in HSM. Such a framework synthesis is achieved as a combination of the relevant framework, theory, or conceptual models and particularly is applied for developing a priori framework based on deductive reasoning [25]. The BFF approach is appropriate to create conceptual models to describe or express the decisions and behaviors of individuals and groups in a particular domain. This is distinct from other methods of evidence synthesis because it employs a systematic approach to create an initial framework for synthesis based on existing frameworks, models, or theories [25] for identifying and adapting theories systematically with the rapid synthesis of evidence [25, 26]. The initial framework can be derived from a relatively well-known model in the target field, or be formed by the integration of several existing models. The initial framework is then reduced to its key components that have shaped its concepts [25]. Indeed, the initial framework considers as the basis and it can be rebuilt, extended, or reduced based on its dimensions [26]. New concepts also emerge based on the researchers' interpretation of the evidence and ongoing comparisons of these concepts across studies [25]. This approach of synthesis possesses both positivist and interpretative perspectives; it provides the simultaneous use of the well-known strengths of both framework and evidence synthesis [27].

In order to achieve this aim the following methodological steps were conducted as follows:

### Searching and selection of studies

In this step, we aimed to look for the relevant models and frameworks related to evidence-based decision-making in health systems management. The main research question was “what is the best framework for EBDM in health systems?” after defining the research question, the researchers searched for published studies on EBDM in HSM in different scientific databases with relevant keywords and constraints as inclusion and exclusion criteria from 01.01.2000 to 12.31.2020 (Table 1).

**Table 1:** Search strategy for the review

**Databases:** ISI web of science, PubMed (PMC, MEDLINE), Scopus, Science Direct, ProQuest, EMBASE	
**Limits:** Language (English), In Title/Abstract (keywords), Full text Available, Document type: Article, Review, Dissertation & Thesis	
**Publication date:** 2000 up to 2020	
**Search strategy:** #1 AND #2 AND #3	
**#1**	"Evidence-Based Decision-Making" OR "Evidence-Based Management" OR "Evidence-Based Policy-Making" OR "Evidence-Informed Decision-Making" OR "Evidence-Informed Policy"
**#2**	Criteria OR Factor* OR Component* OR part* OR element* OR segment* OR item* OR determinant* OR section* OR Process OR Model OR Framework
**#3**	Health OR Hospital*
Example (Scopus database)	( TITLE-ABS-KEY ( *"Evidence-Based Decision-Making"* OR *"Evidence-Based Management"* OR *"Evidence-Based Policy-Making"* OR *"Evidence-Informed Decision-Making"* OR *"Evidence-Informed Policy"*) AND TITLE-ABS-KEY ( Criteria OR Factor* OR Component* OR part* OR element* OR segment* OR item* OR determinant* OR section* OR Process OR Model OR Framework) AND TITLE-ABS-KEY ( *health* OR hospital*) AND LANGUAGE ( *english*)) AND DOCTYPE ( *ar* OR *re*) AND PUBYEAR > *2000*

### Inclusion and exclusion criteria

**Fig. 1:**
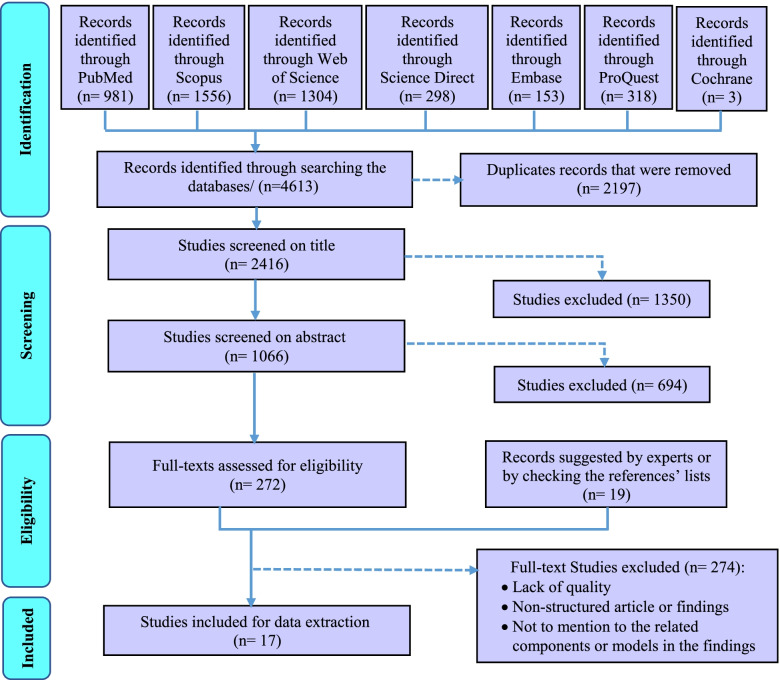
The PRISMA flowchart for selection of the studies in scoping review

Inclusion criteria were determined as the studies that identify the components or develop a model or framework of EBDM in health organization in the form of original or review articles or dissertations, which were published in English and had a full text. The studies like book reviews, opinion articles, and commentaries that lacked a specific framework for conducting our review were excluded. During the search phase of the study, we attempted as much as possible to access studies that were not included in the search process or gray literature by reviewing the references lists of the retrieved studies or by contacting the authors of the articles or experts and querying them, as well as manually searching the related sites (Fig. 1).

### Quality appraisal

The quality of the obtained studies was investigated using three tools for assessing the quality of various types of studies considering types and methods of the final include studies in systematic review. These tools were including Critical Appraisal Skills Program (CASP) for assessing the quality of qualitative researches [28], Scale for the Assessment of Narrative Review Articles (SANRA) [29], and The Mixed Methods Appraisal Tool (MMAT) version 2018 for information professionals and researchers [30] (Table 3-Appendix).

### Data extraction

After searching the studies from all databases and removing duplicates, the studies were independently reviewed and screened by two members (TS and MRAM) of the research team in three phases by the title, abstract, and then the full text of the articles. At each stage of the study, the final decision to enter the study to the next stage was based on agreement and, in case of disagreement, the opinion of the third person from the research team was asked (PB). Mendeley reference manager software was used to systematically search and screen relevant studies. The data from the included studies were extracted based on the study questions and accordingly, a form of the studies’ profile including the author's name, publication year, country, study title, type of study, and its conditions were prepared in Microsoft Excel software (Table 4-Appendix).

### Synthesis and the conceptual model

In this step, a thematic analysis approach was applied to extract and analyze the data. For this purpose, first, the texts of the selected studies were read several times, and the initial qualitative codes or thematic concepts, according to the determined keywords and based on the research question, were found and labeled. Then these initial thematic codes were reviewed to achieve the final codes and they were integrated and categorized to achieve the final main themes and sub-themes, eventually. The main and the sub-themes are representative of the main and sub-steps of EBDM. At the last stage of the synthesis, the thematic analysis was finalized with 8 main themes and all the main and the sub-themes were tabulated (Table 5-Appendix).

### Creation of a new conceptual framework

For BFF synthesis in the present study, we compared the existing models and tried to find a model that fits the best. Three related models that appeared to be relatively well-suited to the purpose of this study to provide a complete, comprehensive, and practical EBDM model in HSM were found. According to the BFF instruction in Carroll et al. (2013) study [25], we decided to use all three models as the basis for the best fit because any of those models were not complete enough and we could give no one an advantage over others. Consequently, the initial model or the BFF basis was formed and the related thematic codes were classified according to the category of this basis as the main themes/steps of EBDM in HSM (Table 5-Appendix). Then, the additional founded thematic codes were added and incorporated to this basis as the other main steps and the sub-steps of the EBDM in HSM according to the research team and some details in the form of sub-steps were added by the research team to complete the synthesized framework. Eventually, a comprehensive practical framework consisting of 10 main steps and 47 sub-steps was created with the potentiality of applying and implementing EDBM in HSM that we categorized them into four main phases (Table 6-Appendix).

### Testing the synthesis: comparison with the a priori models, dissonance and sensitivity

In order to assess the differences between the priori framework and the new conceptual framework, the authors tried to ask some experts’ opinions about the validity of the synthesized results. The group of experts has included eight specialists in the field of health system management or health policy-making. These experts have been chosen considering their previous research or experience in evidence-based decision/policy making performance/management (Table 2). This panel lasted in two three-hour sessions. The finalized themes and sub-themes (Table 6-Appendix) and the new generated framework (Fig. 3) were provided to them before each session so that they could think and then in each meeting they discussed them. Finally, all the synthesized themes and sub-themes resulted were reviewed and confirmed by the experts.

**Table 2:** The demographic characteristic of the experts that participated in the synthesis

Variables		Frequency (percent)
Expertise of experts	health system management	4 (50)
	health policy-making	4 (50)
Gender	Male	6 (75)
	Female	2 (25)
Workplace	Tehran University of medical sciences	2 (25)
	Iran University of medical sciences	2 (25)
	Shiraz University of medical sciences	2 (25)
	Esfahan University of medical sciences	2 (25)
Age (Mean)		47
Work experience (year) (Mean)		10

### Ethical considerations

To prevent bias, two individuals carried out all stages of the study such as screening, data extraction, and data analysis. The overall research project related to this manuscript was approved by the medical ethics conceal of the research deputy of Shiraz University of Medical Sciences with approval number IR.SUMS.REC.1396–01-07–14184, too.

## Results

The initial search across six electronic databases and the Cochrane library yielded 4613 studies. After removing duplicates, 2416 studies were assessed based on their titles. According to the abstract screening of the 1066 studies that remained after removing the irrelevant titles, 291 studies were selected and were entered into the full-text screening phase. Due to full-text screening of the studies, 17 final studies were selected for extracting the components and steps of EBDM in HSM (Fig. 1). The features of these studies were summarized in Table 4-Appendix (see supplementary data). Furthermore, according to the quality appraisal of the included studies, the majority of them had an acceptable level of quality. These results have been shown in Table 3-Appendix.

Results of the thematic analysis of the evidence (Table 5-Appendix) along with the concepts proposed and added by the research team according to the focus-group discussion of the experts were shown in Table 6-Appendix. Accordingly, the main steps and related sub-steps of the EBDM process in HSM were defined and categorized.

**Fig. 2:**
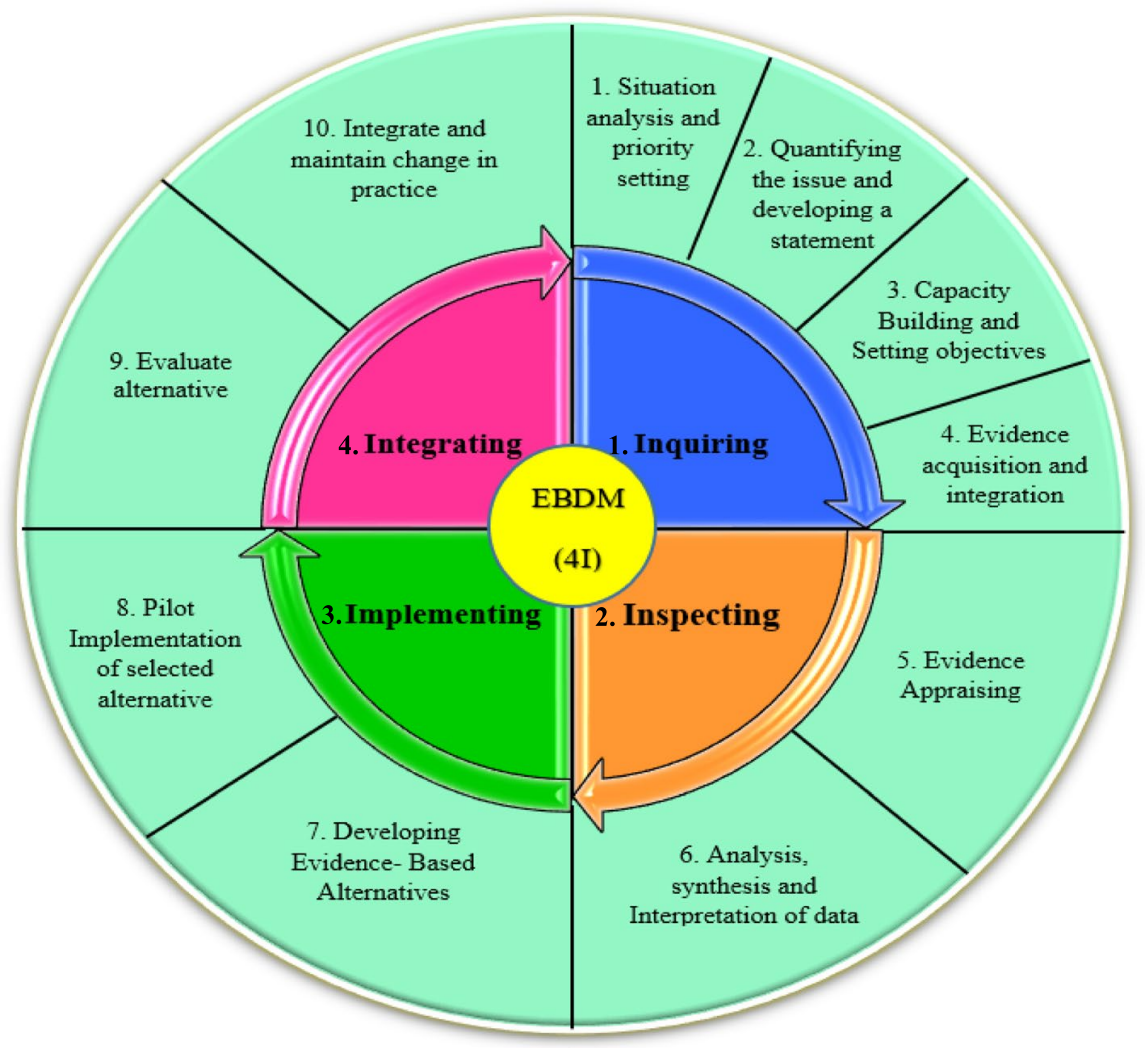
The final synthesized framework of evidence-based decision-making in health system management

**Fig. 3:**
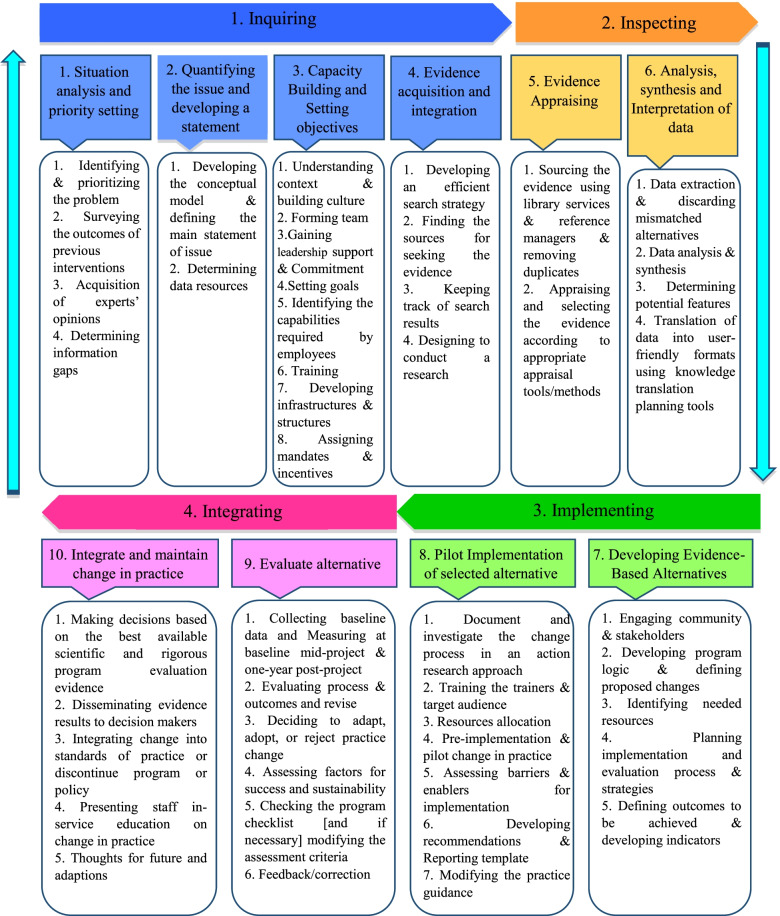
The main steps and sub-steps of the framework of EBDM in health system management

After collecting, synthesizing, and categorizing thematic concepts, incorporating them with the initial models, and adding the additional main steps and sub-steps to the basic models, the final synthesized framework as a best-fit framework for EBDM in HSM was developed in the form of four general phases of inquiring, inspecting, implementing, and integrating and 10 main steps (Fig. 2). For better illustration, this framework with all the main steps and 47 sub-steps has been shown in Fig. 3, completely.

## Discussion

In the present study, a comprehensive framework for EBDM in HSM was developed. This model has different distinguishing characteristics than the formers. First of all, this is a comprehensive practical model that combined the strengths and the crucial components of the limited number of previous models; second, the model includes more details and complementary steps and sub-steps for full implementation of EBDM in health organizations and finally, the model is benefitted from a cyclic nature that has a priority than the linear models. Concerning the differences between the present framework and other previous models in this field, it must be said that most of the previous models related to EBDM were presented in the scope of medicine (that they were excluded from our SR according to the study objectives and exclusion criteria). A significant number of those models were proposed for the scope of public health and evidence-based practice, and only a limited number of them focused exactly on the scope of management and policy/decision making in health system organizations.

Given that the designed model is a comprehensive 10-step model, it can be used in some way at all levels of the health system and even in different countries. However, there will be a difference here, given that this framework provides a practical guide and a comprehensive guideline for applying evidence-based decision-making approach in health systems organizations, at each level of the health system in each country, this management approach can be applied depending on their existing infrastructure and the processes that are already underway (such as capacity building, planning, data collection, etc.), and at the same time, with a general guide, they can provide other infrastructure as well as the prerequisites and processes needed to make this approach much more possible and applicable.

It is true that evidence-based management is different from evidence-based medicine and even more challenging (due to lack of relevant data, greater sensitivity in data collection and their accuracy, lack of consistency and lack of transparency in the implementation of evidence-based decision-making in management rather than evidence-based medicine, etc.). Still, the general framework provided in this article can be used to help organizations that really want to act and move forward through this approach.

Furthermore, based on the findings, most of the previous studies only referred to some parts of the components and steps of the EBDM in health organizations and neglected the other parts or they were not sufficiently comprehensive [31–40]. Most of the previous models did not mention the necessary sub-steps, tools, and practical details for accurate and complete implementation of the EBDM, which causes the organizations that want to use these models, will be confused and cannot fully implement and complete the EBDM cycle. Among the studies that have provided a partly complete model than the other studies, were the studies by Brownson (2009), Yost (2014), and Janati (2018) [3, 41, 42]. Consequently, the combination of these three studies has been used as the initial framework for the best-fit synthesis in the present study.

Likewise, the models presented by Brownson (2009) and Janati (2018) were only limited to the six or seven key steps of the EBDM process, and they did not mention the details required for doing in each step, too [3, 4, 42]. Also, the model presented in the study of Janati (2018) was linear, and the relationships between the EBDM components were not well considered [42, 43]; however, the model presented in this study was recursive. Also, in Yost's study (2014), despite the 7 main steps of EBDM and some details of each of the steps, the proposed process was not schematically drawn in the form of a framework and therefore the relationships between steps and sub-steps were not clear [41]. According to what was discussed, the best-fit framework makes the possibility of concentrating the fragmented models to a comprehensive one that can be fully applied and evaluated by the health systems policymakers and managers.

In the present study, the framework of EBDM in HSM was developed in the form of four general scopes of inquiring, inspecting, implementing, and integrating including 10 main steps and 47 sub-steps. These scopes were discussed as follows:

### Inquiring

In the first step, “situation analysis and priority setting”, the most frequently cited sub-step was identifying and prioritizing the problem. Accordingly, Falzer (2009), emphasized the importance of identifying the decision-making conditions and the relevant institutions and determining their dependencies as the first steps of EBDM [44]. Aas (2012) has also cited the assessment of individuals and problem status and problem-finding as the first steps of EBDM [34]. Moreover, the necessity of identifying the existing situation and issues and prioritizing them has been emphasized as the initial steps in most management models such as environmental analysis in strategic planning [45].

Despite considering the opinions and experience of experts and managers as one of the important sources of evidence for decision-making [42, 46–50], many studies did not mention this sub-step in the EBDM framework. Hence, the present authors added the acquisition of experts’ opinions as a sub-step of the first step because of its important role in achieving a comprehensive view of the overall situation.

In the second step, “quantifying the issue and developing a statement”, “Developing the conceptual model for the issue” was more addressed [37, 41, 47]. In addition, the authors to complete this step added the fourth sub-step, “Defining the main statement of issue”. This is because that most of the problems in health settings may have a similar value for managers and decision-makers and quantifying them can be used as a criterion for more attention or selecting the problem as the main issue to solve.

The third step, “Capacity building and setting objectives”, was not seen in many other included studies as a main step in EBDM, however, the present authors include this as a main step because without considering the appropriate objectives and preparing necessary capacities and infrastructures, entering to the next steps may become problematic. Moreover, in numerous studies, factors such as knowledge and skills of human resources, training, and the availability of the essential structures and infrastructures have been identified as facilitators of EBDM [51–55]. According to this justification, they are included in the present framework as sub-steps of the third step.

Considering the third step and based on the knowledge extracted from the previous studies, the three sub-steps of “understanding context and Building Culture” [56, 57], “gaining the support and commitment of leaders” [39, 57, 58], and “identifying the capabilities required by employees and their skills weaknesses” [58–60] were the most important sub-steps in this step of EBDM framework. In this regard, Dobrow (2004) has also stated that the two essential components of any EBDM are the evidence and context of its use [32]. Furthermore, Isfeedvajani (2018) stated that to overcome barriers and persuade hospital managers and committees to apply evidence-based management and decision-making, first and foremost, creating and promoting a culture of "learning through research" was important [61].

The present findings showed that in the fourth main step, “evidence acquisition and integration”, the most important sub-step was “finding the sources for seeking the evidence” [39–41, 60, 62, 63]. Concerning the sources for the use of evidence in decision-making in HSM, studies have cited numerous sources, most notably scientific and specialized evidence such as research, articles, academic reports, published texts, books, and clinical guidelines [39, 64, 65]. After scientific evidence, using the opinions and experiences of experts, colleagues, and managers [42, 46, 49, 66] as well as the use of census and local level data [49, 66, 67], and other sources such as financial [67], political [42, 49] and evaluations [49, 68] data were cited.

### Inspecting

The fifth step of the present framework, “evidence appraising”, was emphasized by previous literature; for instance, Pierson (2012) pointed to the use of library services in EBDM [69]. Appraising and selecting the evidence according to appropriate appraisal tools/methods was cited the most. International and local evidence is confirmed that ignoring these criteria can lead to serious faults in the process of decision and policy-making [70, 71].

Furthermore, the sixth step, “analysis, synthesis, and interpretation of data”, was mentioned in many included studies [36, 39, 41, 42, 57, 59, 72]. This step emphasized the role of analysis and synthesis of data in the process of generation applied and useful information. It is obvious that the local interpretation according to different contexts may lead to achieving such kind of knowledge that can be used as a basis for local EBDM in HSM.

### Implementing

The third scope consisted of the seventh and eighth steps of the EBDM process in HSM. In the seventh step, “developing evidence-based alternatives”, the issue of involving stakeholders in decision-making and subsequently, planning to design and implementation of the process and evaluation strategies had been focused by the previous studies [58, 60, 62, 63, 73]. Studies by Belay (2009) and Armstrong (2014) had also emphasized the need to use stakeholder and public opinion as well as local and demographic data in decision-making [49, 67].

“Pilot-implementation of selected alternatives” was the eighth step of the framework. Some key sub-steps of this step were resources allocation [58], Pre-implementation and pilot change in practice and assessing barriers and enablers for implementation [40] that indicated the significance of testing the strategies in a pilot stage as a pre- requisition of implementing the whole alternatives. It is obvious that without attention to the pilot stage, adverse and unpleasant outcomes may occur that their correction process imposes many financial, organizational, and human costs on the originations. In addition, a study explained that one of the strategies of the decision-makers to measure the feasibility of the policy options was piloting them, which had a higher chance of being approved by the policymakers. Also, pilot implementation in smaller scales has been recommended in public health in cases of lack of sufficient evidence [74].

### Integrating

This last scope consists of the ninth and tenth steps. The main sub-step of the ninth step, “evaluating alternatives”, was to evaluating process and outcomes and revise. After a successful implementation of the pilot, this step can be assured that the probable outcomes may be achieved and this evaluation will help the decision and policymakers to control the outcomes, effectively. Also, it impacts the whole target program and proposes some correcting plans through an accurate feedback process, too. Pagoto (2007) explained that a facilitator for EBDM would be an efficient and user-friendly system to assess utilization, outcomes, and perceived benefits [55].

Also, the tenth step, “integrating and maintaining change in practice”, was not considered as a major step in previous models, too, while it is important to maintain and sustain positive changes in organizational performance. In this regard, Ward (2011) also suggested several steps to maintain and sustain the widespread changes in the organization, including increasing the urgency and speed of action, forming a team, getting the right vision, negotiating for buy-in, empowerment, short-term success, not giving up and help to make a change stick [35]. Finally, the most important sub-steps that could be mentioned in this step were the dissemination of evidence results to decision-makers and the integration of changes made to existing standards and performance guidelines. Liang (2012) had also emphasized the importance of translating existing evidence into useful practices as well as disseminating them [47]. In addition, the final sub-step, “feedback and feedforward towards the EBDM framework”, was explained by the authors to complete the framework.

Some previous findings showed that about half and two-thirds of organizations do not regularly collect related data about the use of evidence, and they do not systematically evaluate the usefulness or impact of evidence use on interventions and decisions [75]. The results of a study conducted on healthcare managers at the various levels of an Iranian largest medical university showed that the status of EBDM is not appropriate. This problem was more evident among physicians who have been appointed as managers and who have less managerial and systemic attitudes [76]. Such studies, by concerning the shortcomings of current models for EBDM in HSM or even lack of a suitable and usable one, have confirmed the necessity of developing a comprehensive framework or model as a practical guide in this field. Consequently, existing and presenting such a framework can help to institutionalize the concept of EBDM in health organizations.

In contrast, results of Lavis study (2008) on organizations that supported the use of research evidence in decision-making reported that more than half of the organizations (especially institutions of health technology assessment agencies) may use the evidence in their process of decision-making [75], so applying the present framework for these organizations can be recommended, too.

### Limitations

One of the limitations of the present study was the lack of access to some studies (especially gray literature) related to the subject in question that we tried to access them by manual searching and asking from some articles’ authors and experts. In addition, most of the existing studies on EBDM were limited to examining and presenting results on influencing, facilitating, or hindering factors or they only mentioned a few components in this area. Consequently, we tried to search for studies from various databases and carefully review and screen them to make sure that we did not lose any relevant data and thematic code. Also, instead of one model, we used four existing models as a basis in the BFF synthesis so that we can finally, by adding additional codes and themes obtained from other studies as well as expert opinions, provide a comprehensive model taking into account all the required steps and details. Also, the framework developed in this study is a complete conceptual model made by BFF synthesis; however, it may need some localization, according to the status and structure of each health system, for applying it.

## Conclusions

The present framework provides a comprehensive guideline that can be well adapted for implementing EBDM in health systems and organizations especially in underdeveloped and developing countries where there is usually a lag in updating and applying evidence in their decision-making process. In addition, this framework by providing a complete, well-detailed, sequential and practical process including 10 steps and 56 sub-steps that did not exist in the incomplete related models, can be tested in the organizational decision-making process or managerial tasks by developed countries to improve their EBDM cycle, too.

## Data Availability

All data in a form of data extraction tables are available from the corresponding author on a reasonable request.

## Appendix

Tables 3, 4, 5 and 6.

**Table 3:** Quality assessment of the included studies

**Study title**	**Critical** Appraisal Skills Program (CASP) for assessing the quality of qualitative researches (yes/no/can’t tell**)**									
	Was there a clear statement of the aims of the research?	Is a qualitative methodology appropriate?	Was the research design appropriate to address the aims of the research? (Appropriate research design)	Was the recruitment strategy appropriate to the aims of the research? (Sampling)	Were the data collected in a way that addressed the research issue? (Data collection)	Has the relationship between researcher and Participants been adequately considered? (Reflexivity)	Have ethical issues been taken into consideration? (Ethical Issues)	Was the data analysis sufficiently rigorous? (Data Analysis)	Is there a clear statement of findings? (Findings)	How valuable is the research? (Value of the research)
Public Health Decision-Makers’ informational Needs and Preferences for Receiving Research Evidence	Yes	Yes	Yes	Can’t tell	Yes	Yes	Yes	Yes	Yes	Yes
Challenging Evidence-based Decision-making: A Hypothetical Case Study about Return to Work	Yes	Yes	Can’t tell	No	Yes	Can’t tell	Can’t tell	Can’t tell	Yes	Yes
Factors Affecting Evidence-Based Decision Making in Local Health Departments	Yes	Yes	Yes	Yes	Yes	Yes	Can’t tell	Can’t tell	Yes	Yes
Organizational impact of evidence-informed decision making training initiatives: a case study comparison of two approaches	Yes	Yes	Yes	Yes	Yes	Yes	Yes	Yes	Yes	Yes
Tools to support evidence-informed **public health decision making**	Yes	Yes	Yes	Can’t tell	Yes	Can’t tell	Yes	No	Yes	Yes
Evidence-based Management in Practice: Opening up the Decision Process, Decision-maker and Context Evidence-Based	No	Can’t tell	Yes	Yes	Yes	Yes	Can’t tell	Yes	Yes	Yes
Perception of Nursing Middle Managers about the Evidence-Based Management	Yes	Yes	Yes	Can’t tell	Yes	Yes	Yes	Yes	Yes	Yes
Evidence-based management of Caribbean health systems: barriers and opportunities	Yes	Yes	Yes	Yes	Yes	Yes	Can’t tell	Yes	Yes	Yes
An Evidence-Based Framework for Evidence-Based Management in Healthcare Organizations: A Delphi Study	Yes	Yes	Yes	Yes	Yes	Yes	Yes	No	Yes	Yes
Evidence-Based Management Competency Model for Managers in Hospital Settings	Yes	Yes	Yes	Yes	Yes	Yes	Can’t tell	Yes	Yes	Yes
**Study title**	**Scale for the Assessment of Narrative Review Articles (SANRA) (**score from 0 to 2**)**									
	Justification of the article’s importance for the readership	Statement of the concrete aims or formulation of questions	Description of the literature search	referencing	Scientific reasoning	Appropriate presentation of data				
Evidence-Based Public Health: A Fundamental Concept for Public Health Practice	2	1	0	2	1	2				
A framework to improve evidence-informed decision-making in health service management	2	2	1	2	2	2				
**Study title**	**The Mixed Methods Appraisal Tool (MMAT) version 2018 for information professionals and researchers (**yes/no/can’t tell**)**									
	Screening questions		Questions for Mixed methods studies							
	Are there clear research questions?	Do the collected data allow to address the research questions?	Is there an adequate rationale for using a mixed methods design to address the research question?	Are the different components of the study effectively integrated to answer the research question?	Are the outputs of the integration of qualitative and quantitative components adequately interpreted?	Are divergences and inconsistencies between quantitative and qualitative results adequately addressed?	Do the different components of the study adhere to the quality criteria of each tradition of the methods involved?			
Understanding evidence: a statewide survey to explore evidence-informed public health decision-making in a local government setting	Yes	Yes	Yes	Yes	Yes	No	Can’t tell			
Sustainability in Health care by Allocating Resources Effectively (SHARE) 7: supporting staff in evidence-based decision-making, implementation, and evaluation in a local healthcare setting	Yes	Yes	Yes	Yes	Yes	Can’t tell	Can’t tell			
Sustainability in Health care by Allocating Resources Effectively (SHARE) 8: developing, implementing, and evaluating an evidence dissemination service in a local healthcare setting	Yes	Yes	Yes	Yes	Yes	Can’t tell	Can’t tell			
Evaluation of the performance and achievements of the WHO Evidence informed Policy Network (EVIPNet) Europe	Yes	Yes	Yes	Yes	Yes	Can’t tell	Yes			
Promoting the use of evidence in health policymaking in the ECOWAS region: the development and contextualization of an evidence-based policymaking guidance	Yes	Yes	Yes	Yes	No	No	Can’t tell

**Table 4:** Summary of characteristics of included studies

Study number	First Author (Year)	Country	Study Title	Study Design	Setting
1	Maureen Dobbins (2007) [72]	Ontario, Canada	Public Health Decision-Makers’ informational Needs and Preferences for Receiving Research Evidence	Qualitative research (semi-structured interviews)	16 participants (nine program managers, six directors, and one Medical Officer of Health)
2	Ross C. Brownson (2009) [3]	US	Evidence-Based Public Health: A Fundamental Concept for Public Health Practice	Descriptive review	––
3	Zhanming Liang (2012) [47]	Australia	A framework to improve evidence-informed decision-making in health service management	literature review	46 studies were included
4	Randi W. Aas (2012) [34]	Norway	Challenging Evidence-based Decision-making: A Hypothetical Case Study about Return to Work	Qualitative research (case study/ Interviews)	30 participants
5	Collette D. Sosnowy (2013) [54]	New York, USA	Factors Affecting Evidence-Based Decision Making in Local Health Departments	Qualitative research (Interviews, focus groups)	47 participants (7 commissioners, 21 health directors, and 19 other upper-level staff)
6	François Champagne (2014) [36]	6 province of Canada	Organizational impact of evidence-informed decision making training initiatives: a case study comparison of two approaches	Qualitative research (Interviews, documents analysis)	84 participants, organizational documents and organizations’ web sites
7	Rebecca Armstrong (2014) [49]	Victoria, Australia	Understanding evidence: a statewide survey to explore evidence-informed public health decision-making in a local government setting	Mixed-method (cross-sectional survey and interviews)	135 survey respondents and 13 interviews
8	Jennifer Yost (2014) [41]	Ontario, Canada	Tools to support evidence-informed public health decision making	Qualitative research (Interviews)	37 participants
9	April L. Wright (2016) [37]	Australia	Evidence-based Management in Practice: Opening up the Decision Process, Decision-maker and Context Evidence-Based	Qualitative research (case study/ semi-structured interview)	24 emergency physicians and registrars, 4 hospital executives, and 1 nurse
10	Wilza Carla Spiri (2017) [65]	São Paulo, Brazil	Perception of Nursing Middle Managers about the Evidence-Based Management	Qualitative research (Case Study/ individual structured interview)	9 nurse managers
11	Damian Eisenghower Greaves (2017) [77]	Caribbean island	Evidence-based management of Caribbean health systems: barriers and opportunities	Qualitative research (semi-structured interviews)	20 senior managers/leaders (7 ministers for health, 7 permanent secretaries and 6 chief medical officers from 7 countries)
12	Claire Harris (2017) [39]	Australia	Sustainability in Health care by Allocating Resources Effectively (SHARE) 7: supporting staff in evidence-based decision-making, implementation, and evaluation in a local healthcare setting	Mixed methods (Literature reviews, surveys, interviews)	literature review, 178 survey responses and 68 interviews
13	Claire Harris (2018) [40]	Australia	Sustainability in Health care by Allocating Resources Effectively (SHARE) 8: developing, implementing, and evaluating an evidence dissemination service in a local healthcare setting	Mixed methods (Literature reviews, surveys, interviews)	literature review, 164 survey responses and 27 interviews
14	Ali Janati (2018) [42]	Iran	An Evidence-Based Framework for Evidence-Based Management in Healthcare Organizations: A Delphi Study	Qualitative research (Delphi study)	66 participants
15	Louise Lester (2020) [58]	Europe	Evaluation of the performance and achievements of the WHO Evidence informed Policy Network (EVIPNet) Europe	Mixed-method research	21 current Evidence informed Policy Network (EVIPNet) Europe member countries
16	Lina Daouk-Öyry (2020) [60]	Lebanon	Evidence-Based Management Competency Model for Managers in Hospital Settings	Qualitative research (semi-structured interviews)	36 executive managers from 11 hospitals
17	Chigozie Jesse Uneke (2020) [63]	West Africa	Promoting the use of evidence in health policymaking in the ECOWAS region: the development and contextualization of an evidence-based policymaking guidance	Mixed-method research (review and consultations)	15 countries of West African sub-region

**Table 5:** The steps and sub-steps of the EBDM framework resulted from thematic analysis

Steps (frequency of references)	Sub-Steps (frequency of reference)
Situation analysis and priority setting **(7)**	Identifying and prioritizing the problem **(4)**
	Surveying the results of previous interventions **(4)**
	Determining information gaps **(2)**
Quantifying the issue and developing a statement **(2)**	Developing the conceptual model for the issue **(3)**
	Experts’ opinions/experiments **(1)**
Capacity building **(2)**	Understanding the context **(2)** and building an evidence-based culture **(1)**
	Gaining leadership support & commitment **(3)**
	Identifying the capabilities required by employees and their skills weaknesses **(3)**
	Training **(1)**
	Developing the necessary infrastructures and structures **(1)**
	Assigning mandates **(1)** and determining incentives **(2)**
Evidence acquisition and integration **(10)**	Developing an efficient search strategy **(2)**
	Finding the sources for seeking the evidence **(6)** according to 6S Pyramid **(1)** including: Scientific literature **(2)**, Rapid Reviews **(1),** Expert panels **(1),** Patient’s experience **(1),** Professional expertise **(1),** Consultation **(1),** case studies **(1)**
	Keeping track of search results (1)
Evidence appraising **(7)**	Sourcing the evidence **(1)**, library services and reference managers **(1)**
	Appraising and selecting the evidence according to appropriate appraisal tools/methods such as: AGREE II instrument, AMSTAR Tool, Critical Appraisal Skills Program (CASP) Tools, Scottish Intercollegiate Guidelines Network, Quality Assessment Tool for Quantitative Studies **(1),** Benefits & risks, feasibility, applicability, and transferability data **(5)**
Analysis, synthesis and interpretation of data **(7)**	Data extraction **(1)**
	Data analysis and synthesis **(3)** according to: evidence format, style of presentation, accessibility, validity, context sensitivity, applicability, timeliness
	Determining potential features (scope, components, knowledge brokers, target audience, methods) **(1)**
	Translation of data into user-friendly formats **(1)** using knowledge translation planning tools **(1)**
Developing evidence- based alternatives **(8)**	Engaging community and stakeholders **(3)** and participatory decision making **(3)**
	Developing program logic **(1)** and defining proposed change alternatives **(1)**
	Identifying needed resources **(1)**
	Planning implementation and evaluation process and strategies **(1)**
	Defining outcomes to be achieved (29, 30) and develop indicators **(1)**
Pilot implementation of selected alternatives **(10)**	Resources allocation **(1)**
	Pilot change in practice **(1)**
	Assessing barriers and enablers for implementation **(1)**
Evaluate alternative **(8)**	Collecting baseline data **(1)**
	Evaluating processes and outcomes **(2)** and revise **(1)**
	Deciding to adapt, adopt, or reject practice change (**(1)**
	Assessing factors for success and sustainability **(1)**
	Checking the program checklist **(1)**
Integrate and maintain change in practice **(1)**	Disseminating evidence results to decision makers **(2)**, Essential information conveyed effectively to target audiences/stakeholders **(2)**
	Integrating change into standards of practice **(1)** or discontinue program or policy **(1)**
	Thoughts for future and adaptions **(1)**

**Table 6:** The finalized steps and sub-steps of the EBDM framework resulted from evidence synthesis and the research team analysis

Steps	Sub-Steps
Situation analysis and priority setting **(7)**^a^	Identifying and prioritizing the problem **(4)**
	Surveying the results of previous interventions **(4)**
	Acquisition of experts’ opinions **(RTS)**^b^
	Determining information gaps **(2)**
Quantifying the issue and developing a statement **(2)**	Developing the conceptual model for the issue **(3)** and defining the main statement of issues **(RTS)**
	Determining data resources like: surveillance data or clinical problems data **(RTS)**, process improvement or risk-management data **(RTS)**, internal/external benchmarking data **(RTS)**, financial data **(RTS)**, national agencies or organizational standards and guidelines **(RTS)**, new researches and other literature **(RTS)**, and experts’ opinions/experiments **(1)**
Capacity building **(2)** and setting objectives **(RTS)**	Understanding the context **(2)** and building an evidence-based culture **(1)**
	Forming a team **(RTS)**
	Gaining leadership support & commitment **(3)**
	Setting objectives **(RTS)**
	Identifying the capabilities required by employees and their skills weaknesses **(3)**
	Training **(1)**
	Developing the necessary infrastructures and structures **(1)** like improving health information systems **(RTS)**
	Assigning mandates (20) and determining incentives **(2)**
Evidence acquisition and integration **(10)**	Developing an efficient search strategy **(2)**
	Finding the sources for seeking the evidence **(6)** according to 6S Pyramid **(1)** including: Scientific literature **(2),** Meta-analysis or meta-synthesis **(RTS)**, Rapid Reviews **(1),** Other types of evidence (case-report, expert opinion, scientific principles, theory **(RTS)**, Expert panels **(1),** Patient’s experience **(1),** Professional expertise **(1),** Consultation **(1),** Risk assessments **(RTS)**, Economic data **(RTS)**, case studies **(1)**
	Keeping track of search results **(1)**
	(If necessary) designing to^a^ conduct research **(RTS)**
Evidence appraising **(7)**	Sourcing the evidence **(1)** using library services and reference managers **(1)** and Removing duplicates **(RTS)**
	Appraising and selecting the evidence according to appropriate appraisal tools/methods^a^ such as: AGREE II instrument, AMSTAR Tool, Critical Appraisal Skills Program (CASP) Tools, Scottish Intercollegiate Guidelines Network, Quality Assessment Tool for Quantitative Studies **(1),** Benefits & risks, feasibility, applicability, and transferability data **(5)**
Analysis, synthesis and interpretation of data **(7)**	Data extraction **(1)** and discarding mismatched alternatives **(RTS)**
	Data analysis and synthesis **(3)** according to: evidence format, style of presentation, accessibility, validity, context sensitivity, applicability, timeliness
	Determining potential features (scope, components, knowledge brokers, target audience, methods) **(1)**
	Translation of data into user-friendly formats **(1)** using knowledge translation planning tools **(1)**
Developing evidence- based alternatives **(8)**	Engaging community and stakeholders **(3)** and participatory decision making **(3)**
	Developing program logic **(1)** and defining proposed change alternatives **(1)**
	Identifying needed resources **(1)**
	Planning implementation and evaluation process and strategies **(1)** /design EBP guideline(s) **(RTS)**
	Defining outcomes to be achieved **(2)** and develop indicators **(1)**
Pilot implementation of selected alternatives **(10)**	Document and investigate the change process in an action research approach **(RTS)**
	Training the trainers and target audience **(RTS)** and empowerment staff according to the specific needs assessments **(RTS)**
	Resources allocation **(1)**
	Pre-implementation **(RTS)** and pilot change in practice **(1)**
	Assessing barriers and enablers for implementation **(1)**
	Developing recommendations and reporting template **(RTS)**
	Modifying the practice guidance **(RTS)**
Evaluate alternative **(8)**	Collecting baseline data **(1)** and Measuring at baseline mid-project and one-year post-project **(RTS)**
	Evaluating processes and outcomes **(2)** and revise **(1)**
	Deciding to adapt, adopt, or reject practice change **(1)**
	Assessing factors for success and sustainability **(1)**
	Checking the program checklist **(1)** [and if necessary] modifying the assessment criteria **(RTS)**
	Feedback/correction **(RTS)**
Integrate and maintain change in practice **(1)**	Making decisions based on the best available scientific and rigorous program evaluation evidence **(RTS)**
	Disseminating evidence results to decision makers **(2)/** [making sure that] Essential information conveyed effectively to target audiences/stakeholders **(2)**
	Integrating change into standards of practice **(1)** or discontinue program or policy **(1)**
	Presenting staff in-service education on change in practice **(RTS)**
	Thoughts for future and adaptions **(1)/** Feedback and feedforward to evidence-based decision-making model **(RTS)**

^a^ The numbers in parentheses indicates the frequency of references that include the concept

^b^ RTS stands for the concepts synthesized, proposed and added by the research team and confirmed by the experts

## Abbreviations

EBDM: Evidence-based decision-making
HSM: Health System Management
BFF: Best-Fit Framework

## References

[1] Rychetnik L, Bauman A, Laws R, King L, Rissel C, Nutbeam D (2012). Translating research for evidence-based public health: Key concepts and future directions. J Epidemiol Community Health.

[2] Nutbeam D, Boxall AM. What influences the transfer of research into health policy and practice? Observations from England and Australia. Public Health. 2008;122(8):747–53. 10.1016/j.puhe.2008.04.020 18561966

[3] Brownson RC, Fielding JE, Maylahn CM (2009). Evidence-Based Public Health: A Fundamental Concept for Public Health Practice. Annu Rev Public Health [Internet].

[4] Brownson RC, Gurney JG, Land GH. Evidence-based decision making in public health. J Public Heal Manag Pract [Internet]. 1999 [cited 2018 May 26];5(5):86–97. Available from: https://books.google.com/books?hl=en&lr=&id=yxRgAwAAQBAJ&oi=fnd&pg=PA133&dq=+criteria+OR+health+%22evidence+based+decision+making%22&ots=hiqVNQtF24&sig=jms9GsBfw6gz1cN2FQXzCBZvmMQ 10.1097/00124784-199909000-00012 10558389

[5] McGinnis JM. ‘Does Proof Matter? Why Strong Evidence Sometimes Yields Weak Action.’ Am J Heal Promot. 2001;15(5):391–396. Available from: 10.4278/0890-1171-15.5.391. 10.4278/0890-1171-15.5.391 11502031

[6] Majdzadeh R, Yazdizadeh B, Nedjat S, Gholami J, Ahghari S, R. M, et al. Strengthening evidence-based decision-making: Is it possible without improving health system stewardship? Health Policy Plan [Internet]. 2012 Sep 1 [cited 2018 May 15];27(6):499–504. Available from: 10.1093/heapol/czr072 10.1093/heapol/czr072 22027555

[7] WHO EIPNET. Using evidence and innovation to strengthen policy and practice. 2008.

[8] Ellen ME, Léon G, Bouchard G, Lavis JN, Ouimet M, Grimshaw JM (2013). What supports do health system organizations have in place to facilitate evidence-informed decision-making? A qualitative study. Implement Sci..

[9] Oxman AD, Lavis JN, Lewin S, Fretheim A (2009). What is evidence-informed policymaking?. Health Res Policy Sys.

[10] Armstrong R, Waters E, Dobbins M, Anderson L, Moore L, Petticrew M, et al. Knowledge translation strategies to improve the use of evidence in public health decision making in local government: Intervention design and implementation plan. Implement Sci [Internet]. 2013;8(1):1. Available from: https://www.scopus.com/inward/record.uri?eid=2-s2.0-84885076767&doi=10.1186%2F1748-5908-8-121&partnerID=40&md5=dddaf4029205c63877a3e6ecf28f7762 10.1186/1748-5908-8-121 PMC3853093 24107358

[11] Waters E, Armstrong R, Swinburn B, Moore L, Dobbins M, Anderson L, et al. An exploratory cluster randomised controlled trial of knowledge translation strategies to support evidence-informed decision-making in local governments (The KT4LG study). BMC Public Health [Internet]. 2011;11(1):34. Available from: http://www.biomedcentral.com/1471-2458/11/34 10.1186/1471-2458-11-34 PMC3034678 21226966

[12] Imani-Nasab MH, Yazdizadeh B, Salehi M, Seyedin H, Majdzadeh R (2017). Validity and reliability of the Evidence Utilisation in Policymaking Measurement Tool (EUPMT). Heal Res Policy Syst.

[13] El-Jardali F, Lavis JN, Ataya N, Jamal D, Ammar W, Raouf S (2012). Use of health systems evidence by policymakers in eastern mediterranean countries: Views, practices, and contextual influences. BMC Health Serv Res..

[14] Ettelt S, Mays N. Health services research in Europe and its use for informing policy. J Health Serv Res Policy [Internet]. 2011 Jul 29 [cited 2019 Jun 29];16(2_suppl):48–60. Available from: 10.1258/jhsrp.2011.011004 10.1258/jhsrp.2011.011004 21737529

[15] Sutcliffe S. Evidence-Based Policymaking: What is it? How does it work? What relevance for developing countries? 2005.

[16] Campbell DM, Redman S, Jorm L, Cooke M, Zwi AB, Rychetnik L (2009). Increasing the use of evidence in health policy: Practice and views of policy makers and researchers. Aust New Zealand Health Policy.

[17] WHO. Supporting the Use of Research Evidence (SURE) for Policy in African Health Systems. 2007:1–72.

[18] Health Public Accreditation Board. Public Health Accreditation Board STANDARDS : AN OVERVIEW. 2012.

[19] Riley WJ, Bender K, Lownik E (2012). Public health department accreditation implementation: Transforming public health department performance. Am J Public Health.

[20] Liebman JB. Building on Recent Advances in Evidence-Based Policymaking. 2013:36. Available from: http://www.americaachieves.org/9F42769C-3A65-4CCB-8466-E7BFD835CFE7/FinalDownload/DownloadId-0AB31816953C7B8AFBD2D15DFD39D5A8/9F42769C-3A65-4CCB-8466-E7BFD835CFE7/docs/RFA/THP_Liebman.pdf

[21] Jacobs J a, Jones E, Gabella B a, Spring B, Brownson C. Tools for Implementing an Evidence-Based Approach in Public Health Practice. Prev Chronic Dis [Internet]. 2012;9(1):1–9. Available from: 10.5888/pcd9.110324 10.5888/pcd9.110324 PMC3457760 22721501

[22] Kothari A, Edwards N, Hamel N, Judd M (2009). Is research working for you? validating a tool to examine the capacity of health organizations to use research. Implement Sci.

[23] Oxman AD, Bjørndal A, Becerra-Posada F, Gibson M, Block MAG, Haines A, et al. A framework for mandatory impact evaluation to ensure well informed public policy decisions. Lancet. 2010;375(9712):427–31. Available from: 10.1016/S0140-6736(09)61251-4 10.1016/S0140-6736(09)61251-4 20113827

[24] Oxman AD, Vandvik PO, Lavis JN, Fretheim A, Lewin S (2010). SUPPORT Tools for evidence-informed health Policymaking (STP) 2: Improving how your organisation support the use of research evidence to inform policymaking. Chinese J Evidence-Based Med.

[25] Carroll C, Booth A, Leaviss J, Rick J (2013). “ Best fit ” framework synthesis : refining the method. BMC Med Res Methodol.

[26] Carroll C, Booth A, Cooper K (2011). A worked example of “ best fit ” framework synthesis : A systematic review of views concerning the taking of some potential chemopreventive agents. BMC Med Res Methodol.

[27] Barnett-page E, Thomas J. Methods for the synthesis of qualitative research : a critical review. BMC Med Res Methodol 9. 2009;59:1–26. 10.1186/1471-2288-9-59 PMC3224695 19671152

[28] Public Health Resource Unit. Critical Appraisal Skills Programme (CASP) making sense of evidence;10 questions to help you make sense of qualitative research. Public Health. 2006.

[29] Baethge C, Goldbeck-Wood S, Mertens S (2019). SANRA—a scale for the quality assessment of narrative review articles. Res Integr Peer Rev.

[30] Hong Q, Pluye P, Fàbregues S, Bartlett G, Boardman F, Cargo M, et al. Mixed Methods Appraisal Tool (MMAT), Version 2018. User guide. McGill [Internet]. 2018;1–11. Available from: http://mixedmethodsappraisaltoolpublic.pbworks.com/w/file/fetch/127916259/MMAT_2018_criteria-manual_2018-08-01_ENG.pdf%0Ahttp://mixedmethodsappraisaltoolpublic.pbworks.com/

[31] Titler MG. The Iowa Model of evidence-based practice to promote quality care. 2001. 11778337

[32] Dobrow MJ, Goel V, Upshur REG. Evidence-based health policy: context and utilisation. Soc Sci Med [Internet]. 2004;58(1):207–17. Available from: http://www.sciencedirect.com/science/article/pii/S0277953603001667 10.1016/s0277-9536(03)00166-7 14572932

[33] Kohatsu ND, Robinson JG, Torner JC (2004). Evidence-based public health: An evolving concept. Am J Prev Med.

[34] Aas RW, Alexanderson K. Challenging Evidence-based Decision-making: A Hypothetical Case Study about Return to Work. Occup Ther Int. 2012 [cited 2018 May 28];19(1):28–44. Available from: 10.1002/oti.326 10.1002/oti.326 22162107

[35] Ward M (2011). Evidence-informed decision making in a public health setting. Healthc Manag Forum..

[36] Champagne F, Lemieux-Charles L, Duranceau M-F, MacKean G, Reay T. Organizational impact of evidence-informed decision making training initiatives: A case study comparison of two approaches. Implement Sci [Internet]. 2014;9(1). Available from: https://www.scopus.com/inward/record.uri?eid=2-s2.0-84900004312&doi=10.1186%2F1748-5908-9-53&partnerID=40&md5=df5573f17e19ef002d67cf669059aef4 10.1186/1748-5908-9-53 PMC4014624 24885800

[37] Wright AL, Zammuto RF, Liesch PW, Middleton S, Hibbert P, Burke J, et al. Evidence-based Management in Practice: Opening up the Decision Process, Decision-maker and Context. Br J Manag. 2016;27(1):161–78. Available from: 10.1111/1467-8551.12123

[38] Stamatakis KA, Ferreira Hino AA, Allen P, McQueen A, Jacob RR, Baker EA, et al. Results from a psychometric assessment of a new tool for measuring evidence-based decision making in public health organizations. Eval Program Plann [Internet]. 2017 Feb [cited 2018 May 16];60:17–23. Available from: http://linkinghub.elsevier.com/retrieve/pii/S014971891630009X 10.1016/j.evalprogplan.2016.08.002 PMC5140729 27665067

[39] Harris C, Allen K, Waller C, Dyer T, Brooke V, Garrubba M, et al. Sustainability in Health care by Allocating Resources Effectively (SHARE) 7: supporting staff in evidence-based decision-making, implementation and evaluation in a local healthcare setting. BMC Health Serv Res [Internet]. 2017 Dec 21 [cited 2018 May 19];17(1):430. Available from: 10.1186/s12913-017-2388-8 10.1186/s12913-017-2388-8 PMC5480160 28637473

[40] Harris C, Garrubba M, Melder A, Voutier C, Waller C, King R, et al. Sustainability in Health care by Allocating Resources Effectively (SHARE) 8: Developing, implementing and evaluating an evidence dissemination service in a local healthcare setting. BMC Health Serv Res [Internet]. 2018;18(1). Available from: https://www.scopus.com/inward/record.uri?eid=2-s2.0-85042873576&doi=10.1186%2Fs12913-018-2932-1&partnerID=40&md5=2daa5bbd3ccddf299f7b9d527b6105af 10.1186/s12913-018-2932-1 PMC5833068 29499702

[41] Yost J, Dobbins M, Traynor R, DeCorby K, Workentine S, Greco L. Tools to support evidence-informed public health decision making. BMC Public Health [Internet]. 2014 Dec 18 [cited 2018 May 28];14(1):728. Available from: https://bmcpublichealth.biomedcentral.com/articles/10.1186/1471-2458-14-728 10.1186/1471-2458-14-728 PMC4223550 25034534

[42] Janati A, Hasanpoor E, Hajebrahimi S, Sadeghi- H, Khezri A (2018). An Evidence-Based Framework for Evidence-Based Management in Healthcare Organizations : A Delphi Study. Ethiop J Heal Sci.

[43] Rosswurm MA, Larrabee JH (1999). A Model for Change to Evidence-Based Practice. J Nurs Scholarsh.

[44] Falzer PR, Garman MD. A conditional model of evidence-based decision making. J Eval Clin Pract [Internet]. 2009 Dec [cited 2018 May 19];15(6):1142–51. Available from: http://doi.wiley.com/10.1111/j.1365-2753.2009.01315.x 10.1111/j.1365-2753.2009.01315.x PMC2851249 20367718

[45] Ginter PM, Duncan WJ, Swayne LE. Strategic management of health care organizations. 8th Edition. Hoboken: John Wiley & Sons, Inc. 2018. p. 40.

[46] Humphries S, Stafinski T, Mumtaz Z, Menon D. Barriers and facilitators to evidence-use in program management: a systematic review of the literature. BMC Health Serv Res [Internet]. 2014 Dec 14 [cited 2018 May 16];14(1):171. Available from: http://bmchealthservres.biomedcentral.com/articles/10.1186/1472-6963-14-171 10.1186/1472-6963-14-171 PMC4101853 24731719

[47] Liang Z, Howard PF, Leggat SG, Murphy G. A framework to improve evidence-informed decision-making in health service management. Aust Heal Rev [Internet]. 2012;36(3):284–9. Available from: https://www.scopus.com/inward/record.uri?eid=2-s2.0-84865450745&doi=10.1071%2FAH11051&partnerID=40&md5=c42d56774cd486b8df8a5e4afec33a6a 10.1071/AH11051 22935119

[48] Kohn MK, Berta W, Langley A, Davis D. Evidence-based decision making in health care settings: From theory to practice [Internet]. Vol. 11, Advances in Health Care Management. Emerald Group Publishing Ltd; (2011). 215–234 p. Available from:.

[49] Armstrong R, Waters E, Moore L, Dobbins M, Pettman T, Burns C, et al. Understanding evidence: a statewide survey to explore evidence-informed public health decision-making in a local government setting. Implement Sci [Internet]. 2014;9(1):188. Available from: http://implementationscience.biomedcentral.com/articles/10.1186/s13012-014-0188-7 10.1186/s13012-014-0188-7 PMC4314798 25496505

[50] McDiarmid M, Sandra K, Binns M. Evidence-based administrative decision making and the Ontario hospital CEO: information needs, seeking behaviour, and access to sources. JCHLA / JABSC [Internet]. 2007 [cited 2018 May 28];28:63–72. Available from: https://ejournals.library.ualberta.ca/index.php/jchla/article/viewFile/24060/17884

[51] Brownson RC, Allen P, Duggan K, Stamatakis KA, Erwin PC. Fostering more-effective public health by identifying administrative evidence-based practices: A review of the literature. Am J Prev Med [Internet]. 2012;43(3):309–19. Available from: https://www.scopus.com/inward/record.uri?eid=2-s2.0-84865688888&doi=10.1016%2Fj.amepre.2012.06.006&partnerID=40&md5=a843f04e58b619ab9355a98bbfeef51a 10.1016/j.amepre.2012.06.006 PMC3990249 22898125

[52] Moussata CO. Evidence-Based Management and its Influence on the Practices of Senior Leaders of Hospitals in the Denver Metropolitan Area [Internet]. ProQuest Dissertations and Theses. [Ann Arbor]: Colorado Technical University; 2017 [cited 2018 May 19]. Available from: https://search.proquest.com/docview/1967189422?accountid=41313

[53] Ward M, Mowat D. Creating an organizational culture for evidence-informed decision making. Healthc Manag Forum [Internet]. 2012;25(3):146–50. Available from: https://www.scopus.com/inward/record.uri?eid=2-s2.0-84867778644&doi=10.1016%2Fj.hcmf.2012.07.005&partnerID=40&md5=42f06b7236a4a1cd6a4739c7e1900764 10.1016/j.hcmf.2012.07.005 23252330

[54] Sosnowy CD, Weiss LJ, Maylahn CM, Pirani SJ, Katagiri NJ. Factors affecting evidence-based decision making in local health departments. Am J Prev Med [Internet]. 2013 Dec [cited 2018 May 19];45(6):763–8. Available from: http://dx.doi.org/10.1016/j.amepre.2013.08.004 10.1016/j.amepre.2013.08.004 24237920

[55] Pagoto SL, Spring B, Coups EJ, Mulvaney S (2007). Barriers and Facilitators of Evidence-Based Practice Perceived by Behavioral Science Health Professionals.

[56] Weiss L, Sosnowy C, Maylahn C, Katagiri N, Pirani S. Evidence-Based Decision Making in Local Health Departments Evidence-Based Decision Making in Local Health Departments. Front Public Heal Serv Syst Res. 2012;1(3):1–7. 10.1016/j.amepre.2013.08.004 24237920

[57] Liang Z, Howard PF, Leggat SG, Murphy G. A framework to improve evidence-informed decision-making in health service management. Aust Heal Rev [Internet]. 2012;36(3):284–9. Available from: https://www.scopus.com/inward/record.uri?eid=2-s2.0-84865450745&doi=10.1071%2FAH11051&partnerID=40&md5=c42d56774cd486b8df8a5e4afec33a6a 10.1071/AH11051 22935119

[58] Lester L, Haby MM, Chapman E, Kuchenmüller T (2020). Evaluation of the performance and achievements of the WHO Evidence-informed Policy Network (EVIPNet) Europe. Heal Res policy Syst.

[59] Aas RW, Alexanderson K. Challenging evidence-based decision-making: A hypothetical case study about return to work. Occup Ther Int [Internet]. 2012 Mar [cited 2018 May 28];19(1):28–44. Available from: 10.1002/oti.326 10.1002/oti.326 22162107

[60] Daouk-Öyry L, Sahakian T, van de Vijver F (2020). Evidence-Based Management Competency Model for Managers in Hospital Settings. Br J Manag.

[61] Isfeedvajani MS. Evidence-Based Management and its Application in the Hospital Management Process. Hosp Pract Res [Internet]. 2018 [cited 2018 May 28];3(2):35–6. Available from: http://jhpr.ir/article_60533_8313dd6afa580ef2afa689e6984c8844.pdf

[62] Armstrong R, Waters E, Moore L, Dobbins M, Pettman T, Burns C, et al. Understanding evidence: a statewide survey to explore evidence-informed public health decision-making in a local government setting. Implement Sci [Internet]. 2014 Dec 14 [cited 2018 May 28];9(1):188. Available from: 10.1186/s13012-014-0188-7 10.1186/s13012-014-0188-7 PMC4314798 25496505

[63] Uneke CJ, Sombie I, Johnson E, Uneke BI, Okolo S. Promoting the use of evidence in health policymaking in the ECOWAS region: the development and contextualization of an evidence-based policymaking guidance. Global Health [Internet]. 2020;16(1):73. Available from: http://www.embase.com/search/results?subaction=viewrecord&id=L632558076&from=export 10.1186/s12992-020-00605-z PMC7409627 32762759

[64] Janati A, Hasanpoor E, Hajebrahimi S, Sadeghi-Bazargani H. Health care managers’ perspectives on the sources of evidence in evidence-based hospital management: A qualitative study in Iran. Ethiop J Health Sci [Internet]. 2017;27(6):659–68. Available from: https://www.ajol.info/index.php/ejhs/article/view/162555 10.4314/ejhs.v27i6.11 PMC5811945 29487475

[65] Spiri WC, Kurcgant P, Pereira MV. Perception Of Nursing Middle Managers About The Evidence-Based Management. Int Arch Med [Internet]. 2017;10(February):11. Available from: http://imedicalsociety.org/ojs/index.php/iam/article/view/2311

[66] Harris C, Allen K, Waller C, Green S, King R, Ramsey W (2017). Sustainability in Health care by Allocating Resources Effectively (SHARE) 5: developing a model for evidence-driven resource allocation in a local healthcare setting. BMC Health Serv Res.

[67] Belay T, Mbuya N, Rajan V. Data utilization and evidence-based decision making in the health sector: survey of three Indian states [Internet]. 2009 [cited 2018 May 26]. Available from: https://openknowledge.worldbank.org/handle/10986/3161

[68] Niedzwiedzka BM. Barriers to evidence-based decision making among Polish healthcare managers. Heal Serv Manag Res [Internet]. 2003 May 21 [cited 2018 May 19];16(2):106–15. Available from: 10.1258/095148403321591429 10.1258/095148403321591429 12803950

[69] Peirson L, Ciliska D, Dobbins M, Mowat D. Building capacity for evidence informed decision making in public health: A case study of organizational change. BMC Public Health [Internet]. 2012;12(1):137. Available from: https://www.scopus.com/inward/record.uri?eid=2-s2.0-84857132983&doi=10.1186%2F1471-2458-12-137&partnerID=40&md5=68e18844316881fb3f2662477825d441 10.1186/1471-2458-12-137 PMC3305606 22348688

[70] Buffett C, Ciliska D, Thomas H. Can I Use This Evidence in my Program Decision? Assessing Applicability and Transferability of Evidence. National Collaborating Centre for Methods and Tools (NCCMT) School of Nursing, McMaster University. 2007:1–22.

[71] Buffet C, Ciliska D, Thomas H. It worked there. Will it work here? A tool for assessing applicability and transferability of evidence (A: When considering starting a new program). National Collaborating Centre for Methods and Tools (NCCMT) School of Nursing, McMaster University. 2011;(905):1–2.

[72] Dobbins M, Jack S, Thomas H, Kothari A (2007). Public health decision-makers’ informational needs and preferences for receiving research evidence. Worldviews Evidence-Based Nurs.

[73] Wright AL, Zammuto RF, Liesch PW, Middleton S, Hibbert P, Burke J, et al. Evidence-based Management in Practice: Opening up the Decision Process, Decision-maker and Context. Br J Manag [Internet]. 2016 Jan [cited 2018 May 28];27(1):161–78. Available from: 10.1111/1467-8551.12123

[74] Imani-Nasab MH, Seyedin H, Yazdizadeh B, Majdzadeh R (2017). A Qualitative Assessment of the Evidence Utilization for Health Policy-Making on the Basis of SUPPORT Tools in a Developing Country. Int J Heal Policy Manag.

[75] Lavis JN, Paulsen EJ, Oxman AD, Moynihan R (2008). Evidence-informed health policy 2-Survey of organizations that support the use of research evidence. Implement Sci..

[76] Bastani P, Kavosi Z, Alipoori S, Imani-Nasab MH (2017). Evidence- based Policy and Decision-Making among Health Managers: A Case of Shiraz Univer- sity of Medical Sciences. GMJ.

[77] Greaves DE. Evidence-based management of Caribbean health systems: barriers and opportunities. Int J Health Governance. 2017;22(2):104–117.

